# Unconventional valley-dependent optical selection rules and landau level mixing in bilayer graphene

**DOI:** 10.1038/s41467-020-16844-y

**Published:** 2020-06-10

**Authors:** Long Ju, Lei Wang, Xiao Li, Seongphill Moon, Mike Ozerov, Zhengguang Lu, Takashi Taniguchi, Kenji Watanabe, Erich Mueller, Fan Zhang, Dmitry Smirnov, Farhan Rana, Paul L. McEuen

**Affiliations:** 1 Kavli Institute at Cornell for Nanoscale Science, Ithaca, NY 14853 USA; 2 Laboratory of Atomic and Solid State Physics, Cornell University, Ithaca, NY 14853 USA; 3 Department of Physics, City University of Hong Kong, Kowloon, Hong Kong SAR; 4 National High Magnetic Field Laboratory, Tallahassee, FL 32312 USA; 5 National Institute for Materials Science, 1-1 Namiki, Tsukuba, 305-0044 Japan; 6 Department of Physics, University of Texas at Dallas, Richardson, TX 75080 USA; 7 School of Electrical and Computer Engineering, Cornell University, Ithaca, NY 14853 USA; 8 Present Address: Department of Physics, Massachusetts Institute of Technology, Cambridge, MA 02139 USA

**Keywords:** Materials science, Nanoscience and technology, Optics and photonics, Physics

## Abstract

Selection rules are of vital importance in determining the basic optical properties of atoms, molecules and semiconductors. They provide general insights into the symmetry of the system and the nature of relevant electronic states. A two-dimensional electron gas in a magnetic field is a model system where optical transitions between Landau levels (LLs) are described by simple selection rules associated with the LL index *N*. Here we examine the inter-LL optical transitions of high-quality bilayer graphene by photocurrent spectroscopy measurement. We observed valley-dependent optical transitions that violate the conventional selection rules Δ|*N*| = ± 1. Moreover, we can tune the relative oscillator strength by tuning the bilayer graphene bandgap. Our findings provide insights into the interplay between magnetic field, band structure and many-body interactions in tunable semiconductor systems, and the experimental technique can be generalized to study symmetry-broken states and low energy magneto-optical properties of other nano and quantum materials.

## Abstract (web summary)

Optical transitions between Landau levels (LL) in solids are described by selection rules associated with the LL index. Here, the authors perform photocurrent spectroscopy measurements on high-quality bilayer graphene to investigate the interband LL transitions, and observe valley-dependent optical transitions obeying unusual selection rules.

## Introduction

Bilayer graphene (BLG) has emerged as a two-dimensional semiconductor where the bandgap is tunable by an external electric field. At zero magnetic field, the bandgap hosts strong exciton resonances^1^ that obey unusual optical selection rules determined by the electron pseudospin texture. At large magnetic fields, two-dimensional electron gas is expected to form quantized energy levels that were named after Landau^2^. Properties of individual Landau levels (LLs) have been studied extensively ever since the birth of graphene using electron transport^3–7^, scanning tunneling spectroscopy^8^, and electronic compressibility measurements^9^. Inter-LL optical transitions in graphene could provide direct information of the critical energy scales of SU(4) isospin^6^ degeneracy lifting through spectroscopy and the wave functions of LLs through selection rules. For semiconductor physics, BLG provides a model system where the interplay between band dispersion, cyclotron energy, and Coulomb energy of electron-hole pairs can be systematically studied through gating and tuning magnetic field. However, inter-LL transitions in BLG and the crossover from exciton-dominated to LL-dominated optical response was largely unexplored, let alone the optical selection rules of them.

Previous infrared absorption spectroscopy studies of MLG (both on SiO_2_/Si^10,11^ and on hBN^12,13^), BLG on SiO_2_/Si substrates^14^, and thin graphene layers on SiC^15,16^ suffered from broad peak width or the lack of gating. And they revealed only optical transitions obeying the conventional Δ|*N* | = ± 1 selection rule. Further investigations on higher quality graphene samples, however, have been challenging owing to limited sizes of hBN-encapsulated graphene samples compared with the relevant infrared wavelength. Here we overcome this difficulty by using Fourier Transformed Infrared photocurrent spectroscopy^1^ to study high-quality hBN-encapsulated BLG devices. In addition to greatly enhanced signal-to-noise ratio, such spectroscopy method avoids the background signal owing to absorption in the gates—providing clean data from the layer of interest. Although photocurrent signal is more complicated than optical absorption, it would not affect the main conclusion of this article as the conversion efficiency should be similar for optical transitions with similar energies. As a result, we observed spectral features with a linewidth of ~1 meV and many optical transitions that violate the conventional selection rule^10,12,14,17–19^ Δ|*N*| = ± 1.

## Results

### Overview of (magneto-)photocurrent spectra of BLG

**Fig. 1:**
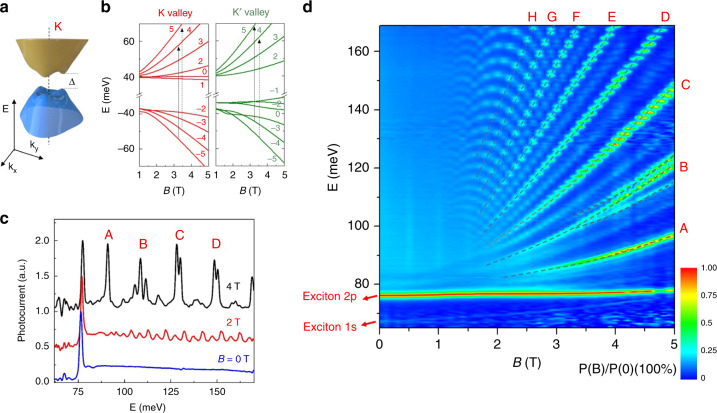
Landau level spectroscopy of band-gapped bilayer graphene (BLG). **a** Illustration of the trigonal-warped BLG band structure with a bandgap Δ in the K valley. **b** Evolution of Landau level energies calculated by a continuum model with Δ = 80 meV. We use red and green colors to label K and K′ valleys, respectively, throughout the paper. Solid and dashed arrows illustrate inter-LL transitions obeying the usual selection rule of Δ|*N*| = ± 1. **c** Photocurrent spectra with a displacement field of *D* = 0.874 V/nm at *B* = 0, 2, and 4 T. The 0 T spectrum features two exciton peaks near the bandgap and a flat spectrum above the bandgap. Oscillations corresponding to inter-LL transitions emerge at 2 T with an alternated high-low pattern. The contrast between high and low peaks increases at 4 T, where splittings of peaks also appear. **d** A 2D color plot of photocurrent spectra as the magnetic field is continuously tuned. Dashed curves trace high peaks in **c**, which grow stronger towards higher magnetic fields and we label them by branch A-H (only branch A-D are shown in **c**). These strong transitions obey the usual optical selection rule of Δ |*N*| = ± 1. The low peaks in **c** evolve into weak transitions between dashed lines, whose optical selection rules will be discussed later.

Figure 1a shows the band structure of BLG near the K point. Around the electric field-induced bandgap *Δ* both conduction and valence bands develop three pockets^20,21^ owing to the trigonal warping effect^22^. Figure 1b presents LL spectra in BLG calculated based on a continuum model with a bandgap of 80 meV (optimized for the spectrum at *D* = 0.874 V/nm, see Supplementary Discussion), where each LL is labeled by its orbital index *N*. Owing to the interlayer potential, LL with *N* = 0 and 1 have a valley-dependent distribution. Together with electron-hole asymmetry inherent in the band structure, inter-LL transitions in K and K′ valleys (especially those close to the bandgap) are expected to have strong differences. Electric-dipole-allowed optical selection rules dictates that initial and final states are different by ± *ħ* in angular momentum, corresponding to that of circularly polarized photons. In ordinary semiconductors, this angular momentum conservation is satisfied by interband transitions at zero magnetic field. Therefore, interband LL transitions are allowed only when Δ|*N*| = 0 as no additional angular momentum change is induced by LL wave functions. In graphene systems^10,12,14^, transition metal dichalcogenides^17^ and other Dirac electron systems^18,19^, however, an extra pseudospin degree of freedom introduces an effective angular momentum for electrons. As a result, the conventional optical transition selection rules were shown to be Δ|*N*| = ± 1 both theoretically and experimentally^10,12,14,23^ as illustrated by solid and dashed arrows in Fig. 1b.

Our hBN-BLG-hBN sample is similar to that used in ref. ^1^. Figure 1c presents photocurrent spectra at a displacement field *D* = 0.874 V/nm with the Fermi level tuned into the bandgap (see ref. ^1^ for the measurement technique). At *B* = 0 T, the spectrum features two sharp peaks, which were previously identified as excitons^1^. Above excitons, the spectrum starts as a flat line and gradually develops oscillations with an increasing *B* field. At 2 T, the oscillations show a pattern of alternating higher and lower peaks. The contrast between these two groups of peaks is enhanced at *B* = 4 T. At the same time, both higher and lower peaks split into finer structures (the latter will be shown more clearly in following figures). These spectra present the continuous evolution of the system behavior from exciton-dominated picture to inter-LL-dominated picture.

To better visualize the evolution of all transition peaks, we plot spectra as a 2D color map in Fig. 1d as *B* is continuously tuned. High peaks in Fig. 1c evolve into branches in the diagonal direction as traced by the dashed curves. We label these branches by A-H. The lower peaks in Fig. 1b evolve into branches between the dashed curves. The contrast in intensity of these two groups of branches increases as *B* rises. We will call them strong and weak transitions hereafter.

### Properties of strong inter-LL transitions

**Fig. 2:**
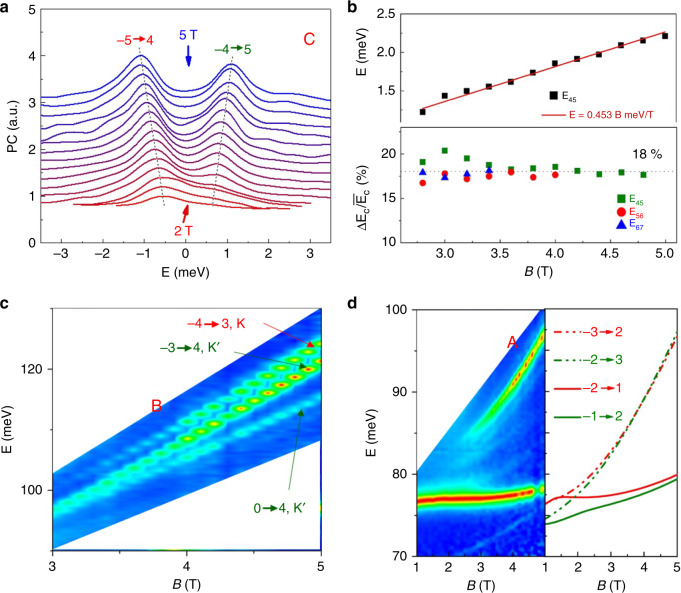
Properties of the strong transitions at *D* = 0.874 V/nm. **a** Evolution of branch C transitions. For clarity, photocurrent spectra at 2–5 T (0.2 T step) are shifted horizontally to the center and vertically by multiples of 0.2. Dashed lines trace transitions corresponding to solid arrows in Fig. 1b. Transitions corresponding to dashed arrows in Fig. 1b are too weak to be identified. **b** Upper panel: the splitting between two peaks in **a**, E_45_. It measures the difference between electron and hole cyclotron energies, ΔE_c_, which scales linearly with B. Lower panel: the ratio between ΔE_c_ and the average cyclotron energy $\overline {E_c}$, indicating an asymmetry of ~18% between electron and hole masses. **c** Evolution of branch B, which is special among all branches as it involves three transitions. The extra transition corresponds to transition (0 → 4, K′). Such Δ|*N*| = ±4 transitions are allowed by the trigonal warping effect. **d** Evolution of the 2p exciton. Left panel: Fig. 1d near and below branch A. Right panel: calculated dispersions of LL transitions. When LLs are not well-established at low magnetic fields, the strong 2p exciton dominates the spectrum. At higher fields, the 2p exciton evolves into a magneto-exciton, whose dispersion is very close to LL transitions (−2 → 1, K) and (−1 → 2, K′).

Discrete peaks in photocurrent spectrum correspond to optical transitions between LLs and Fig. 1d reveals a rich collection of spectral information. To better understand the origins of various transitions, we first focus on branch C as shown in Fig. 2a. We shifted spectra horizontally to align the center of transition peaks to zero energy. This plot clearly shows a pair of peaks as traced by dashed lines, which split more as *B* increases.

By comparing to calculated spectra (see Supplementary Discussion 1B), we identify these two peaks as inter-LL transitions (−5 → 4, K) and (−4 → 5, K′) that correspond to solid arrows in Fig. 1b. Note that our calculation does not include electron-hole interaction, so excitonic effects are not shown. Transitions indicated by dash arrows in the same energy range as in Fig. 1b are found to be much weaker by calculations. These four transitions (two represented by solid lines and two represented by dashed lines) form a quartet that obey the conventional optical selection rule of Δ|*N* | = ± 1. To the first order, the splitting between transitions (−5 → 4, K) and (−4 → 5, K′) reflects the difference between electron and hole cyclotron energies^24^. The upper panel of Fig. 2b shows that the difference Δ*E*_*c*_ scales linearly *B* (the red line). The lower panel of Fig. 2b shows the relative electron-hole asymmetry Δ*E*_*c*_ */*$\bar E_c$, where $\overline {E_c}$ is the averaged cyclotron energy of electron and hole. The extracted ratio of ~18% is close to previously reported asymmetry in the effective masses of electrons and holes^24^. Most strong transition branches behave similarly to branch C (see Supplementary Figure 4).

The only exception is branch B, where three peaks are involved as shown in Fig. 2c. A comparison with our calculation (see Supplementary Figure 3) shows that the extra peak corresponds to (0 → 4, K′), which is forbidden by the simple selection rules Δ|*N*| = ± 1. This is a manifestation of the trigonal warping effect in BLG lattice^22^, which distorts the rotational symmetric Mexican hat band structure to a three-fold rotational symmetric structure as shown in Fig. 1a. Therefore LLs differed by 3 *m* (*m* is an integer) are coupled by this lattice potential, resulting in an extra ±3 ingredient to the selection rule^25–29^. This effect results in inter-LL transitions with Δ|*N*| = ± 4, ±2. Branch B is the only one that has three peaks with similar energies and oscillator strength involved, owing to the close energies of LL 0 and −3 in the K′ valley as shown in Fig. 1b. Ultimately this is owing to the relatively flat band at the top of the conduction band in K′ valley. As the LL index increases, energies of LLs are well separated so Δ|*N*| = ± 4 transitions are far from Δ|*N*| = ± 1 transitions.

Having understood the origin of branch B, we conclude that branch A corresponds to transitions (−3 → 2, K) and (−2 → 3, K′). LL transitions (−1 → 2, K) and (−2 → 1, K′) are also allowed by selection rules and should appear at energy below that of branch A, as shown by our calculation in Fig. 2d. However, the 2p exciton peak is in the same energy range and it persists down to zero magnetic field. As the magnetic field increases, single-particle cyclotron energy gradually surpasses the exciton binging energy. One would expect the nature of this resonance to gradually change from exciton to inter-LL transition^17,30^ as the magnetic increases. At high enough magnetic field, the dispersion of this resonance can be almost described by that of the inter-LL transition, as has been shown in ref. ^30^.

### Properties of weak inter-LL transitions

**Fig. 3:**
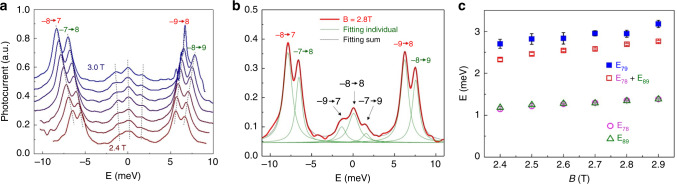
Properties of the weak transitions at *D* = 0.874 V/nm. **a** Evolution of a typical branch of weak transitions between branch F and G in Fig. 1d. Spectra at 2.4–3.0 T (0.1 T step) are shifted horizontally to the center, and vertically by multiples of 0.1. Three peaks emerge gradually in the middle. **b** Fitting of the 2.8 T spectrum using seven Lorentzian peaks. The two side peaks in the weak transition branch correspond to (−9 → 7) and (−7 → 9). These Δ|N| = ± 2 transitions are allowed by the trigonal warping effect. The central peak corresponds to transition (−8 → 8) and its selection rule of Δ|*N*| = 0 is beyond an ideal single-particle picture. **c** Energy splitting between different pairs of peaks indicated by dashed lines in **b**. The splitting within branch F and G are plotted in pink (E_78_) and green (E_89_), respectively, whereas the sum of them is plotted in red (twice of the difference between electron and hole cyclotron energies). The splitting between (−9 → 7) and (−7 → 9) (E_79_ plotted in blue) is very close to E_78_ + E_89_—corroborating our assignment of peaks in **b**.

Next we examine the weak transitions. Figure 3a shows one branch of such transitions between strong transition branch F and G in the range of 2.4–3.0 T with a step size of 0.1 T. The spectra are shifted to align the center of weak transition peaks to zero energy. Interestingly, three peaks emerge from the single peak at low *B* and their splitting widens with increased *B*. Figure 3b shows a fitting of the spectrum at 2.8 T, where the sum of seven Lorentzian lineshaped peaks with an offset describes the experimental spectrum nicely. The linewidths of all peaks are ~1 meV, indicating a very low disorder-induced inhomogeneous broadening in our samples. Figure 3c plots the energy splitting of three pairs of peaks extracted from the fitting in Fig. 3b: *E*_*78*_ and *E*_*89*_ are the splittings within branches F and G; *E*_*79*_ represents the splitting between the two side peaks in the branch of weak transitions. We found that *E*_*79*_ is within 13% of *E*_*78*_ + *E*_*89*._ This observation, together with the fact that weak transition peaks sit at halfway between branches F and G, indicate that the three weak transitions correspond to (−9 → 7, K & K′), (−8 → 8, K & K′), and (−7 → 9, K & K′) as labeled in Fig. 3b. Other branches of weak transitions behave similarly and more fittings can be found in Supplementary Figure 6. We therefore observed inter-LL transitions obeying selection rules of Δ|*N*| = ± 2 and Δ|*N*| = 0. The former can be understood with the same trigonal warping effect as a combination of Δ|*N*| = ± 1 and ±3. But the Δ|*N*| = 0 transitions goes beyond the single-particle picture in intrinsic BLG.

The weak transitions acquire oscillator strengths through LL mixing by either the trigonal warping Hamiltonian or the exciton binding energy (as we will discuss later). As the magnetic field increases, such mixing effect is weakened owing to larger LL separation—cyclotron energy. As a result, these weak transitions diminish relative to the strong transitions towards higher magnetic fields, agreeing with our observations in Fig. 1c, d.

### Relative oscillator strength of inter-LL transitions

**Fig. 4:**
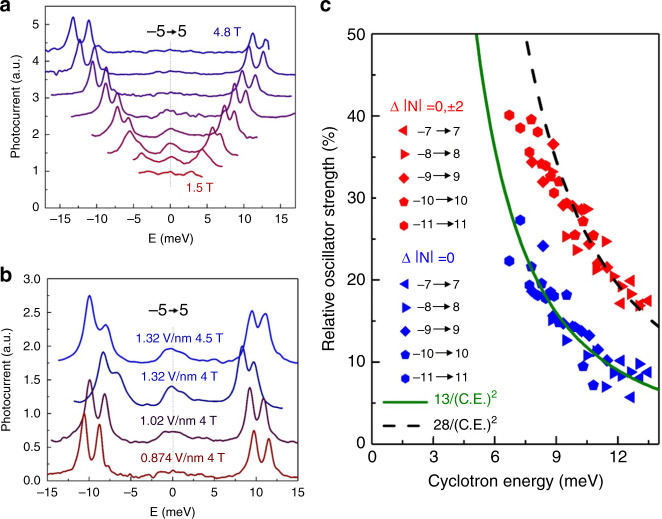
Relative oscillator strength of weak transitions and Δ|*N*| = 0 transitions. **a** Photocurrent spectra in the vicinity of transition (−5 → 5) at *D* = 0.874 V/nm. Spectra at 1.5, 2.0, 2.5, 3.0, 3.5, 4.0, 4.5, 4.8 T are shifted horizontally to the center, and vertically by multiples of 0.6. The contrast between the “strong” and “weak” transitions is negligible at low fields but is appreciable at high fields. At 4.8 T, weak transitions are negligible in the spectrum. **b** Spectra in the vicinity of transition (−5 → 5) at *D* = 0.874, 1.02, and 1.32 V/nm. The relative oscillator strength of weak transitions to strong transitions increases at higher *D* for the same magnetic field of 4 T. Even in the scenario where cyclotron energies are similar (1.32 V/nm & 4.5 T, and 0.874 V/nm & 4 T), the oscillator strength is still more concentrated in weak transitions at higher *D*. **c** Relative oscillator strength as a function of the cyclotron energy at *D* = 1.32 V/nm. Data in blue (red) represent the ratio between the oscillator strength in Δ|*N*| = 0 (Δ|*N*| = 0 & Δ|*N*| = ± 2) transition and the integrated oscillator strength. The relative oscillator strength in Δ|*N*| = 0 is proportional to 1/(C.E.)2, where C.E. is the cyclotron energy. A typical energy scale of LL mixing ~1.8 meV can be extracted from this plot.

We further explore quantitatively the oscillator strength of weak transitions and the Δ|*N*| = 0 transitions relative to the integrated oscillator strength of all transitions. Owing to the complex nature of photocurrent and photoconductivity process and their unknown quantum efficiencies, we limit our discussion to relative oscillator strengths of adjacent transition peaks and avoid extracting the absolute optical conductivity. Figure 4a plots spectra in the vicinity of the (−5 → 5, K & K′) transition at 1.5–4.8 T. For clarity, we shifted spectra horizontally to align the center of transition peaks to zero energy. At 1.5 T, the “weak” and “strong” transitions are of similar peak height and oscillator strength. And the only way to differentiate them is to trace transitions from spectra at higher magnetic fields. As we increase *B*, the central weak transitions gradually weaken compared to strong transitions on both sides. At 4.8 T, weak transitions drop to the noise level of the spectrum. We further explore this contrast in oscillator strength at different bandgaps. Figure 4b shows spectra in the vicinity of (−5 → 5, K & K′) at *D* = 0.874, 1.02, 1.32 V/nm with the same *B* = 4 T. Clearly, more oscillator strength is concentrated in the central weak transitions for a bigger bandgap. We can further quantify the oscillator strength based on fittings as shown in Fig. 3b. Figure 4c plots the oscillator strength of weak transitions (the sum of Δ|*N*| = 0 and ±2 in red, Δ|*N* | = 0 in blue) divided by the total oscillator strength of all transitions. The data from different branches are plotted with different legends at *D* = 1.32 V/nm. To provide a reference for the decreasing trend of relative oscillator strength versus cyclotron energy, we plot a solid green curve and a dashed black curve—both are inversely proportional to the squared cyclotron energy.

## Discussion

The observation of Δ|*N*| = 0 transitions can be qualitatively explained by a simple model in which adjacent inter-LL transitions are mixed by certain mechanism (represented by a mixing Hamiltonian *H*_*m*_). For example, we consider optical transitions (−*N*→*N*), (−*N*→*N* − 1), and (−*N* − 1→*N*) in K valley and label the excited states as |−*N*, *N*>, |−*N*, *N* − 1>, and |−*N* − 1, *N*>, respectively. With the perturbation by *H*_*m*_, the state |−*N*, *N* > changes into |−*N*, *N*>′ = |−*N*, *N* + *a*|−*N*, *N* − 1> +*b*|−*N* − 1, *N*>, where the coefficients *a* and *b* describe the mixing of excited states. The oscillator strength of optical transition from the ground state |0> to |−*N*, *N*>′ is therefore *T* = |′<−*N*, *N*|*H*|0>|^2^ = |*a* < −*N*, *N* − 1|*H*|0 > + *b* < −*N* − 1, *N*|*H*|0>|^2^, where *H* is the electron–photon coupling Hamiltonian. Here the conventional selection rule of Δ|*N*| = ± 1 is included as < −*N*, *N*|*H*|0> = 0. With finite *a* and *b*, transition (−*N*→*N*) could appear in our spectrum as the system is excited from |0> to |−*N*, *N* > ′.

In the first order perturbation theory, the wave function mixing coefficient between two adjacent excited states |−*N*, *N*>, and |−*N* − 1, *N*> is *a*, *b* ~ *E*_*m*_*/E*_*c*_, where *E*_*m*_ = |<−*N*, *N*|*H*_*m*_|−*N*−1, *N*>| is the typical energy scale of the coupling Hamiltonian *H*_*m*_, whereas *E*_*c*_ is the cyclotron energy. Therefore, the oscillator strength of Δ|*N*| = 0 transitions relative to the integrated oscillator strength is |*a* + *b*|^2^ = 4(*E*_*m*_*/E*_*c*_)^2^. Qualitatively, (*E*_*m*_*/E*_*c*_)^2^ decreases as *B* increases as long as *E*_*m*_ follows a sublinear relation with *B*, agreeing with the trend we observed in Fig. 4. Quantitatively, the green curve in Fig. 4c indicates a wave function mixing coefficient of *a*~*b* ~ (1.8 meV/*E*_*c*_) and *E*_*m*_ ~ 1.8 meV.

With this energy scale of the mixing Hamiltonian, we discuss possible microscopic mechanisms. Beyond the single-particle picture in ideal BLG, two possible scenarios could lead to our observation of Δ|*N*| = 0 transitions. In the first scenario, we include disorders but neglect the electron-hole interaction. Such disorder potential could enable the forbidden optical transitions^31^. Experimentally, disordered induced unconventional inter-LL optical transition were indeed observed in GaAs quantum wells^32^ and Raman spectrum of graphene-like domains on graphite^33^. Quantitatively^31^, the forbidden transition has a relative oscillator strength of ~$\left( {{\it{\Gamma /{\rm{E}}}}_c} \right)^2$ with respect to the Δ|*N*| = ± 1 transition, where *Γ* is the scattering induced broadening of a single LL and it plays the role of *E*_*m*_. Quantitatively, by setting 4(*E*_*m*_*/E*_*c*_)^2^ = $\left( {{\it{\Gamma /{\rm{E}}}}_c} \right)^{\mathrm{2}}$ to explain our data, we can obtain *Γ* = 2*E*_*m*_ ~ 3.6 meV. Fitting of the single transition peak in our spectra indicates a Lorentzian linewidth of ~1 meV, which corresponds to 4*Γ* (two for initial and final states, whereas another two for the full width at half-maximum). Therefore, the realistic level broadening ${\it{\Gamma }} \sim 0.25\;\mathrm{meV}$ is more than one order of magnitude smaller than that is needed to explain the observed oscillator strength—indicating possible mechanisms beyond the single-particle picture.

In the second scenario, we include electron-hole interaction. In a general context, interband optical excitations of a 2DEG in magnetic field is described by magneto-excitons^34^. Owing to angular momentum conservation, optically active modes of magneto-excitons at *k* = 0 do not mix^34^. However, modes at finite *k* are results of mixing between |−*N*, *N*> and |−*N*−1, *N*> and the mixing coefficient *a* (*b*) could be significant at *kl*_*B*_~1. Here *l*_*B*_ is the magnetic length defined as *l*_*B*_ = $\sqrt {\hbar /eB}$ ~26 nm/$\sqrt {B(\mathrm{Tesla})}$. These modes can be optically excited when the translational symmetry is broken by disorder or the Moire superlattice. The latter provides a momentum *k*$\ge 2\pi /14 \, \mathrm{nm}$, where 14 nm is the largest period in graphene/hBN Moire superlattice. At 1 Tesla, *kl*_*B*_ is ~10, easily satisfying the required condition for mixing |−*N*, *N*> and |−*N* − 1, *N*>. From the energy point-of-view, the Coulomb energy between electron and hole is expected to play an important role, as the exciton binding energy (~10 meV at *D* = 0.874 V/nm) is big enough compared with 1.8 meV to explain the data. The binding energy is expected to increase with enhanced displacement field and giving forbidden transitions more oscillator strength. This picture is supported by our data in Fig. 4b, where the relative oscillator strength of Δ|*N*| = 0 transitions at *D* = 1.32 V/nm is much larger than that at *D* = 0.874 V/nm. Further theoretical calculation of this specific semiconductor system is needed to corroborate this second scenario.

Our results present the first measurement of inter-LL transitions in high-quality BLG with a tunable bandgap. The many observations provide a plethora of information about interplay between the band structure, magnetic field, and many-body interactions. There is no theoretical prediction of the behavior of Δ|*N*| = 0 transitions in BLG, or its dependence on the bandgap. The selection rule of inter-LL transitions could also be used to study exotic interaction-driven ground states of materials such as the nematic phase^35^, where the optical selection rules can be changed by the broken rotational symmetry. On the other hand, the (magneto-) photocurrent spectroscopy presented here could be extended to study the integer and fractional quantum Hall states in high-quality graphene devices, shining light on the mechanism of valley and spin degeneracy lifting and excitations such as the magneto-rotons^36^. Beyond the quantum Hall systems, our method is particularly suitable for studying strongly correlated electron physics in engineered van der Waals heterostructures of 2D materials^37–41^, e.g., the Moire gap and correlated insulating gaps in twisted graphene/TMD systems as well as ABC-stacked trilayer graphene/hBN superlattices.

## Methods

### Measurement scheme

The basic sample configuration and measurement scheme are similar to that in ref. ^1^. The data at magnetic fields higher than 5 Tesla was collected at the SCM3 in the National High Magnetic Field Laboratory (NHMFL). Sample temperature in the Cornell setup is ~10 K, whereas that in the NHMFL setup is ~5 K owing to the helium exchange gas environment. A Bruker IFS 66 v spectrometer was used at NHMFL. The illumination is focused by a ZnSe lens located outside (inside) the cryostat at Cornell (NHMFL). The thickness of hBN flakes is ~20 nm for both top and bottom gates. All the measurement is done at a total charge density of zero to minimize the dark current noise. The displacement field is calculated by using a dielectric constant of boron nitride of 3.9. The data in this manuscript are mostly based on two separate high-quality graphene samples.

## Supplementary information

Supplementary Information

## Data Availability

The data that support the findings of this study are available from the corresponding author upon request.
