# Author Correction: Unconventional valley-dependent optical selection rules and landau level mixing in bilayer graphene

**DOI:** 10.1038/s41467-020-17168-7

**Published:** 2020-06-24

**Authors:** Long Ju, Lei Wang, Xiao Li, Seongphill Moon, Mike Ozerov, Zhengguang Lu, Takashi Taniguchi, Kenji Watanabe, Erich Mueller, Fan Zhang, Dmitry Smirnov, Farhan Rana, Paul L. McEuen

**Affiliations:** 1 Kavli Institute at Cornell for Nanoscale Science, Ithaca, NY 14853 USA; 2 Laboratory of Atomic and Solid State Physics, Cornell University, Ithaca, NY 14853 USA; 3 Department of Physics, City University of Hong Kong, Kowloon, Hong Kong SAR; 4 National High Magnetic Field Laboratory, Tallahassee, FL 32312 USA; 5 National Institute for Materials Science, 1-1 Namiki, Tsukuba, 305-0044 Japan; 6 Department of Physics, University of Texas at Dallas, Richardson, TX 75080 USA; 7 School of Electrical and Computer Engineering, Cornell University, Ithaca, NY 14853 USA; 8 Present Address: Department of Physics, Massachusetts Institute of Technology, Cambridge, MA 02139 USA

**Keywords:** Materials science, Nanoscience and technology, Optics and photonics, Physics

Correction to: *Nature Communications* 10.1038/s41467-020-16844-y, published online 10 June 2020.

The original version of this Article contained errors in the Acknowledgements:

F.Z. is supported by ARO under Grant No. W911NF-18-1-0416 and NSF under Grant No. DMR-1921581 (DMREF). This was incorrectly given as F.Z. is supported by the University of Texas at Dallas research enhancement funds.

Funding from the NSF MRSEC program (DMR-1719875) was incorrectly given as Funding from the NSF MRSEC program (DMR-1120296).

This has now been corrected in both the PDF and HTML versions of the Article.

