# Supplementary material for: Unconventional valley-dependent optical selection rules and landau level mixing in bilayer graphene
Source: Nat Commun. 2020 Jun 10;11:2941. doi: 10.1038/s41467-020-16844-y (PMC7287093; doi:10.1038/s41467-020-16844-y)
Supplement: Supplementary file 1 — Supplementary Information [file 41467_2020_16844_MOESM1_ESM.pdf]

Supplementary Information

Unconventional Valley-Dependent Optical Selection Rules and Landau Level Mixing in Bilayer Graphene

Long Ju[1,2*#], Lei Wang[1,2*], Xiao Li[3], Seongphill Moon[4], Mike Ozerov[4], Zhengguang Lu[4], Fan Zhang[5], Takashi Taniguchi[6], Kenji Watanabe[6], Dmitry Smirnov[4], Erich Mueller[2], Farhan Rana[6] and Paul L. McEuen[1,2]†

[1]Kavli Institute at Cornell for Nanoscale Science, Ithaca, NY 14853, USA.

[2]Laboratory of Atomic and Solid State Physics, Cornell University, Ithaca, NY 14853, USA.

[3]Department of Physics, City University of Hon Kong, Kowloon, Hong Kong SAR
[4]National High Magnetic Field Laboratory, Tallahassee, Florida 32312, USA

[5]Department of Physics, University of Texas at Dallas, Richardson, TX 75080, USA

[6]National Institute for Materials Science, 1-1 Namiki, Tsukuba 305-0044, Japan.

[7]School of Electrical and Computer Engineering, Cornell University, Ithaca 14853, New York, USA.

# Current Address: Department of Physics, Massachusetts Institute of Technology, Cambridge, MA, 02139, USA

Discussion: Calculation of the optical absorption spectrum of BLG inter-LL transitions

We provide some theoretical backgrounds on our calculations of the LL structure and the optical absorption spectrum of inter-LL transitions in bilayer graphene (BLG). The low-energy effective four- band Hamiltonian for bilayer graphene is given by[1]

$$H_{BLG} = \tau_z \begin{pmatrix} u/2 & v_3\pi & v_4\pi^\dagger & v_0\pi^\dagger \\ v_3\pi^\dagger & -u/2 & v_0\pi & v_4\pi \\ v_4\pi & v_0\pi^\dagger & -u/2 & \tau_z\gamma_1 \\ v_0\pi & v_4\pi^\dagger & \tau_z\gamma_1 & u/2 \end{pmatrix} \quad (1)$$

where $\pi \equiv p_x + ip_y$, and $\pi^\dagger = p_x - ip_y$. In addition, $\tau_z = \pm 1$ labels the two valleys in BLG, and u represents the potential energy differences between the two layers. The various effective velocities that appear in the above model are related to the respective hopping energies (denoted by γ_i) as $v_i = 3a\gamma_i/2\hbar$, where a is the lattice constant of the graphene lattice. Also note that the basis of the above Hamiltonian is different between the two valleys. For $\tau_z = +1$, the basis states

are $\{A_1, B_2, A_2, B_1\}$, where A_1 and A_2 denote the A sublattice on the top and bottom layer, respectively. In contrast, for $\tau_z = -1$, the basis states are $\{B_2, A_1, B_1, A_2\}$ instead.

The effects of a perpendicular magnetic field can be taken into account via the Peierls substitution $\boldsymbol{p} \to \boldsymbol{p} + e\boldsymbol{A}/c$, where $\boldsymbol{A} = (0, Bx)$ is the vector potential. As a result, the operator $\pi = \pi_x + i\pi_y$ coincides with the lowering operators, satisfying $\pi\phi_n = -i\left(\frac{\hbar}{\ell_B}\right)\sqrt{2n}\phi_{n-1}$ and $\pi\phi_0 = 0$. Here $\ell_B = \sqrt{\hbar c/(eB)}$ is the magnetic length and ϕ_n is the nth LL eigenstate of an ordinary two-dimensional electron gas. In the absence of the trigonal warping term ($\gamma_3 = 0$) the LL structure can be obtained easily in a closed form. However, when $\gamma_3 \neq 0$ the dimension of the Hamiltonian matrix becomes infinite, and we have to introduce a cutoff in the Landau level index ($n{\sim}100$) that is high enough to obtain the low-energy spectrum properly[2]. Examples of the LL spectrum are shown in Fig. 1(b) in the maintext. Note that all numerical results in this work are based on the four-band model in Eq. (1).

A. Determination of the model parameters

To determine the model parameters for the BLG sample in our experiment, we compare the experimental photocurrent spectrum with our numerical simulations using the usual Kubo formula approach, and search for an optimal set of parameters. Note that our numerical calculations did not explicitly account for the electron-electron interactions that may be important. As a result, excitonic effects in the absorption spectrum will not show up in our simulations. Since such effects are particularly strong near the band edge, we only compare our simulations with the high energy parts of the spectrum. With that said, the parameters we find are not exactly the same as the familiar Slonczewski-Weiss-McClure (SWM) model of graphite lattice[3], indicating that renormalizations by electron-electron interactions still manifest themselves in our simulations. In particular, we do not force one set of parameters to fit for all magnetic fields, because the relative importance of the electron-electron interactions can depend on the applied magnetic field[4]. Supplementary Figure 1 shows our fitting results for the photocurrent spectrum at 5 T, and the corresponding parameters are summarized in Table ST1. We found that the experimental spectrum can be well-reproduced by the theoretical calculation. Differences between theoretic and experimental appear at energies corresponding to the $\Delta|N| = 0$ transitions, since our tight-binding calculation does not include

possible many-body interactions and the resulted Landau level mixing. This difference, however, is very minor at 5 T when the oscillator strength of $\Delta|N| = 0$ transitions almost diminish.

B. Splitting of the $\Delta|N| = \pm 1$ transitions

We now discuss how to understand various features in the absorption spectrum based on the model in Eq. (1). We start with the splitting of the $\Delta|N| = \pm 1$ transitions. It is well known that dominant inter-LL transitions in bilayer graphene follow the selection rule of $\Delta|N| = \pm 1$, where $\Delta|N|$ denotes the absolute value of LL index difference between the initial and final states. As a result, the main transitions can be grouped into a set of quartets, each containing the following four transitions: $|-N \to N+1, K\rangle$, $|-(N+1) \to N, K\rangle$, $|-N \to N+1, K'\rangle$, and $|-(N+1) \to N, K'\rangle$. For convenience, we call each such quartet the $(N, N+1)$ quartet. We would like to understand how the structure of each quartet depends on the parameters of the BLG model. In the simplest model when $\gamma_3 = \gamma_4 = 0$, all inter-LL transitions are four-fold degenerate. To demonstrate this, we consider the following two-band effective Hamiltonian for bilayer graphene[1],

$$H_{2-band} = -\frac{1}{2m}\begin{pmatrix} 0 & (\pi^\dagger)^2 \\ \pi^2 & 0 \end{pmatrix} + h_w + h_4 + h_{\mathrm{U}} \tag{2}$$

$$h_w = v_3\begin{pmatrix} 0 & \pi \\ \pi^\dagger & 0 \end{pmatrix}, h_4 = \frac{2vv_4}{\gamma_1}\begin{pmatrix} \pi^\dagger\pi & 0 \\ 0 & \pi\pi^\dagger \end{pmatrix}, h_U = \frac{-u}{2}\left[\begin{pmatrix} 1 & 0 \\ 0 & -1 \end{pmatrix} - \frac{2v^2}{\gamma_1^2}\begin{pmatrix} \pi^\dagger\pi & 0 \\ 0 & -\pi\pi^\dagger \end{pmatrix}\right],$$

which can be derived from the four-band model in Eq. (1) in the low-energy limit. In the above Hamiltonian h_w is responsible for trigonal warping effects, while the h_4 creates an electron-hole asymmetry in the band structure. When $v_3 = v_4 = 0$, the energies of LLs can be derived from this two-band model as

$$E_{N,\tau_z} = \frac{1}{2}\left[-\tau_z u\epsilon_0 \pm \sqrt{4N(N-1)\epsilon_0^4 + u^2[(2N-1)\epsilon_0^2 - 1]^2}\right] \tag{3}$$

where we have introduced $\epsilon_0 \equiv \sqrt{2}\hbar v_0/(\gamma_1 l_B)$. It follows immediately that all four transitions in each (N, N+1) quartet remain degenerate. The same conclusion holds for the four-band model in Eq. (1), although and analytical expression for the LL energies is more difficult to obtain.

When the electron-hole asymmetry is present ($v_4 \neq 0$), each quartet splits into two groups: $|-N \to N+1, K\rangle$ and $|-N \to N+1, K'\rangle$ remain degenerate, while $|-(N+1) \to N, K\rangle$ and $|-(N+1) \to N, K'\rangle$ have different energies. Only when both the trigonal warping term and electron-hole asymmetry term are present the four transitions in the quartet split completely.

Supplementary Figure 2 shows the comparison between theoretical and experimental absorption spectrum at a magnetic field strength of 3.8 T. It includes two sets of main transitions: the (5,6) quartet and the (6,7) quartet. We see that each quartet seems to have only two peaks, in contrast to the theoretical predictions above. This is because the four transitions in each quartet have very different optical weights. For example, we find that in the (5,6) quartet the two peaks correspond to the transition |-6→5, K> and |-5→6, K'>, respectively. The other two transitions in this quartet (labeled by 'A1' and 'A2'), however, have very small optical weights, and thus do not show up as major peaks in the absorption spectrum.

C. The appearance of minor transitions due to the trigonal warping effect

Supplementary Figure 2 also shows minor transitions obeying $\Delta|N| = \pm 2, \pm 4$. These transitions can be attributed to the trigonal warping effect of the BLG band structure[5]. Especially, Fig. 2c in the maintext includes a transition |0→4, K'> whose oscillator strength is big enough to make branch B very different from other branches of strong transitions.

Here we provide more details of this transition. Supplementary Fig. 3 shows the evolution of transition energies in branch B as a function of the magnetic field. We noticed that there is a critical field of ~4.2 T at which the order of transitions |-3→4, K'> and |0→4, K'> switches. This is related to the correct assignment of LL indices to eigenstates. In the absence of trigonal warping effects ($\gamma_3 = 0$), the Hamiltonian in Eq. (1) can be solved analytically, and each eigenstate has the following form,

$$\Psi_N^T = \left[A_N \phi_N \quad B_N \phi_{N-2} \quad C_N \phi_{N-1} \quad D_N \phi_{N-1} \right] \tag{4}$$

As a result, each LL can be associated with a particular harmonic oscillator state, and the assignment of LL index is straightforward: we simply track the harmonic oscillator state that appears in the first component of the wave function. When $\gamma_3 \neq 0$, however, the situation becomes more complicated, because the dimension of the Hamiltonian matrix now becomes infinite, and the eigenstates are no longer associated with a single harmonic oscillator state. Instead, we need to expand each component of the wave function as an infinite superposition of different harmonic oscillator states as follows,

$$\Psi_N^T = \left[\sum_n A_n^{(N)} \phi_n, \sum_n B_n^{(N)} \phi_n, \sum_n C_n^{(N)} \phi_n, \sum_n D_n^{(N)} \phi_n \right]$$

where the summation is from n = 0 to +∞. As a result, the original eigenvalue problem is then recast as the eigenvalues of an infinite-dimensional matrix of the superposition coefficients. In practice, however, we introduce a summation cutoff that is large enough to obtain the low-energy LL's properly.

Given such a situation, we will use the index n of the dominant coefficient $A_n^{(N)}$ as an operational definition of the LL index, which converges to the original definition in Eq. (4) when $\gamma_3 = 0$ (in which case we have $A_n^{(N)} = \delta_{N,n}$). In particular, for strong magnetic fields (B > 5 T), the dominant coefficient is independent of the field, and thus allows for a unique identification of the LLs in the presence of trigonal warping effects[6]. In order to avoid unnecessary clusters in notation, we will omit the superscript (N) in the coefficient (which differentiates different LL's) in our discussions below, and only focus on the subscripts n (which differentiates different harmonic oscillator components in each LL).

Under this convention, we can sort all coefficients A_i (i = 0, 1…… N) into three groups according to the remainder when we divide their index i by 3: $\{A_{3k}\} \equiv \{A_0, A_3, ...\}$, $\{A_{3k+1}\} \equiv \{A_1, A_4, ...\}$ and $\{A_{3k+2}\} \equiv \{A_2, A_5, ...\}$, where k is a natural number. The reason for this organization is that for a given eigenstate in this problem, only one group of $\{A_i\}$ will be nonzero (for all magnetic fields), while the other two groups vanish identically. In particular, for the |0, K'> and |-3, K'> LLs in Fig. 1(b) in the maintext, only coefficients in the group $\{A_{3k}\}$ will be nonzero. Moreover, for fields between 3T and 4 T, A_3 is the dominant coefficient for the |0, K'> LL while A_0 is the dominant coefficient for the |-3, K'> LL. In contrast, for fields between 4T and 5 T, A_0 (A_3) is the dominant coefficient for the |0, K'> (|-3, K'>) LL instead.

Such a situation is summarized in Supplementary Fig. 3, where we use color to track the dominant coefficient in the corresponding LL. We can now explain the switching of transitions in Supplementary Fig. 3: for 3T < B < 4 T, the |-3, K'> LL has A_0 as the dominant coefficient in the wave function, and thus the |-3→4, K'> transition in Fig. 2(c) in the maintext is dominated by the dipole optical weight between the ϕ_0 and ϕ_4 harmonic oscillator states, and this is a weak transition, only allowed by the trigonal warping effects. Meanwhile, the |0, K'> LL has A_3 as the dominant coefficient in the wave function, and thus the optical weight for the |0→4, K'> transition is dominated by the dipole optical weight between the ϕ_3 and ϕ_4 harmonic oscillator states, which

has a large optical weight. This is the reason why the |0→4, K'> transition looks brighter than the |-3→4, K'> transition in Fig. 2(c) in the maintext when 3T < B < 4 T. When 4T < B < 5 T, however, the situation is reversed, and the |-3→4, K'> transition looks brighter. Now that we have understood the |0→4, K'>transition, one may wonder where its counterpart, the |-4→0, K> transition is located. In fact, the latter transition has a much smaller frequency, and is located near the (2, 3) quartet, as shown in Supplementary Fig. 4.

Discussion: Original photocurrent spectra that were used generate Fig. 1c in the maintext

In Supplementary Fig. 5, we plot individual photocurrent spectra in the range of 1-5 T with a step size of 0.1 T. These data were used to generate the 2D color plot in Fig. 1c in the maintext.

Discussion: Examples of extracting the oscillator strength from experimental photocurrent spectra

In Supplementary Fig. 6, we show three examples other than that in Fig. 3b in the main text. In each plot, the experimental photocurrent spectrum can be well described by the sum of seven Lorentzian peaks. The linewidth of all the Lorentzian peaks are ~1 meV.

Discussion: Photocurrent spectra at *D* = 1.32 V/nm

In Supplementary Fig. 7, we show the 2D color plot of photocurrent spectra at D = 1.32 V/nm. Features in this figure are qualitatively the same as that in Fig. 1d in the maintext.

Supplementary Figures and Tables

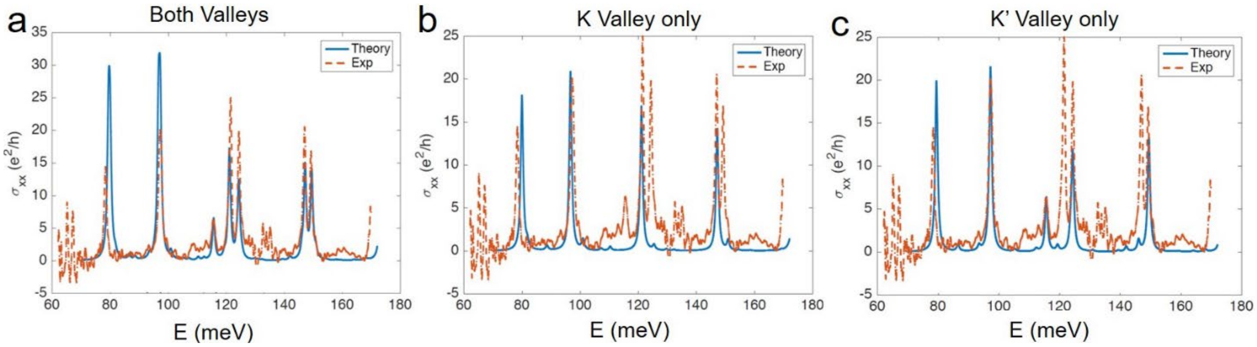

Supplementary Fig. 1 Comparison between experimental photocurrent spectrum and calculated optical conductivity spectra at a *B* = 5 T and *D* = 0.874 V/nm. a-c. The calculated optical conductivity spectrum of both valleys, only the K valley and only the K' valley respectively. Experimental photocurrent spectrum is plotted in all three panels for comparison. The calculated spectra are based on an optimal set of model parameters listed in Table ST1. We focused on the main transition peaks corresponding to $\Delta|N| = \pm 1$ and |0→4, K'>. Note that the trigonal warping effect mostly induced by γ_3 is needed to fit the data. Other smaller transitions almost disappear at 5 T so we do not consider their influence on the fitting. The valley splitting of major transition peaks are clearly seen, supporting our assignment of the peaks in photocurrent spectra.

	γ_0 (eV)	γ_1 (eV)	γ_3 (eV)	γ_4 (eV)	u (eV)	η (meV)
Value	3.2	0.32	0.32	0.256	0.08	0.5

Supplementary Table 1. The set of model parameters we find for the bilayer graphene model in Eq. (1). Here η is the disorder broadening we introduced in our calculations. These parameters are close to reported by previous literatures.

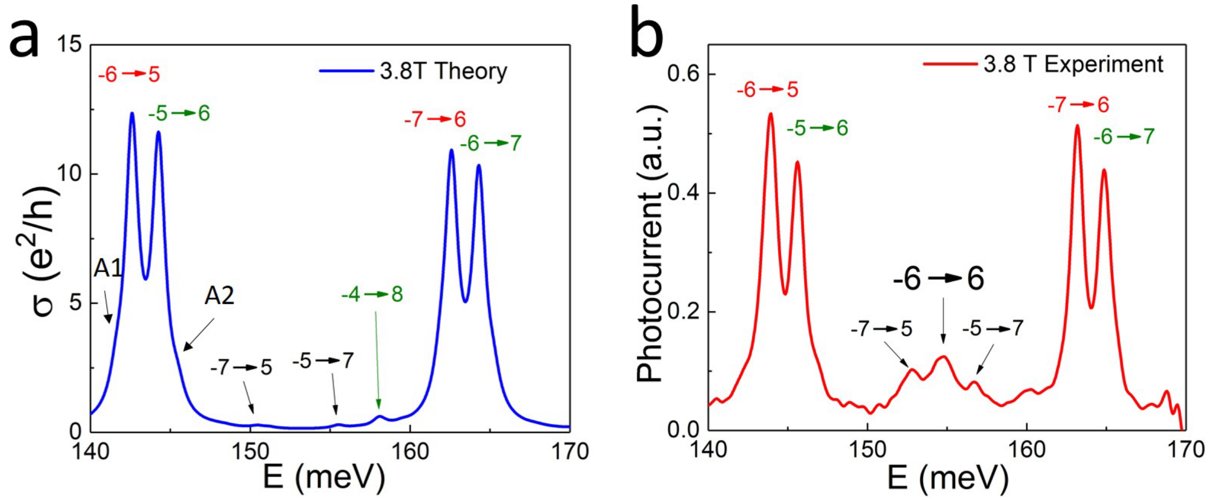

Supplementary Fig. 2 Comparison of theoretical and experimental absorption spectrum at B = 3.8 T and D = 0.874 V/nm. The numerical results in (a) are generated by using parameters in Table ST1 above. In addition to valley polarized transitions obeying $\Delta|N| = \pm 1$, valley degenerate transitions of $\Delta|N| = \pm 2$ are seen in both (a) and (b). We can also see transition |-4→8, K'> obeying $\Delta|N| = \pm 4$ in this range. A1 and A2 indicate the two transitions in the (5,6) quartet which has negligible oscillator strength. Finally, note that the $\Delta|N| = 0$ transitions are completely absent from our numerical simulations.

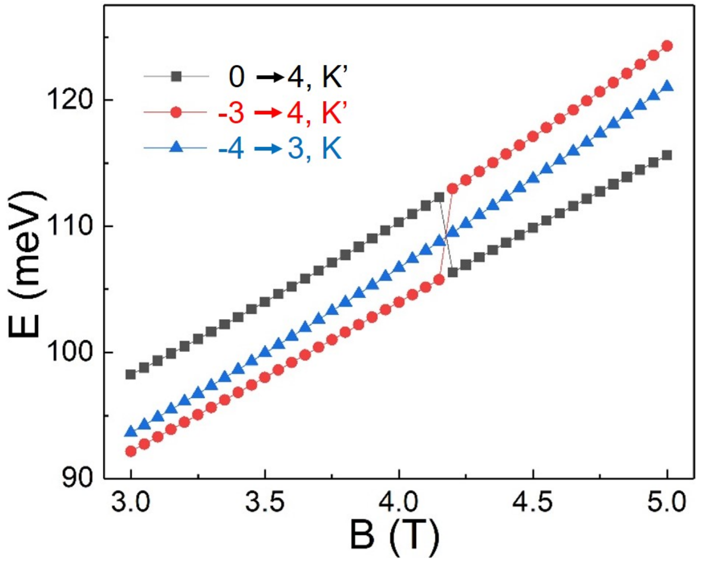

Supplementary Fig. 3 Peaks in the range of the (3,4) quartet calculated using parameters in Table ST1. At 4.2 T, the lowest and highest energy transitions switches to reflect the change of LL in index assigned to eigenstates.

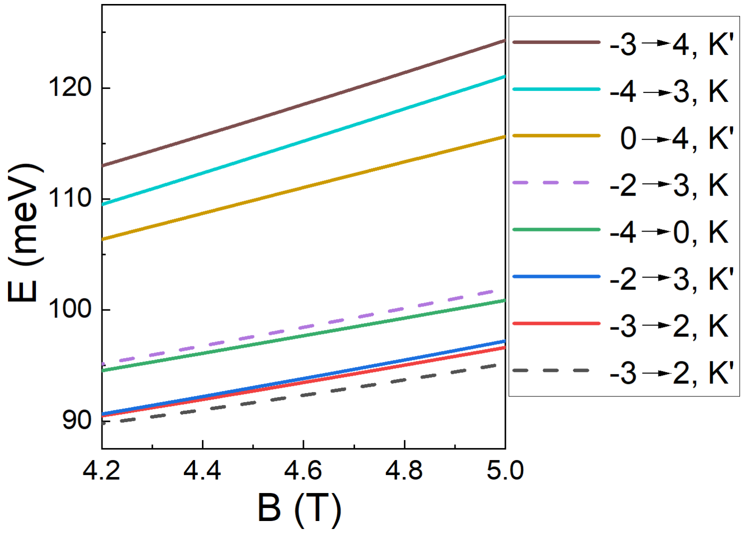

Supplementary Fig. 4 Numerical simulations of the absorption spectrum peaks in the range *B* = 4.2 - 5 T. This result is also obtained using parameters in Table ST1. The |-4→0, K> transition is much closer to transitions in branch A than branch B, agreeing with our experimental observation of only 3 peaks in branch B.

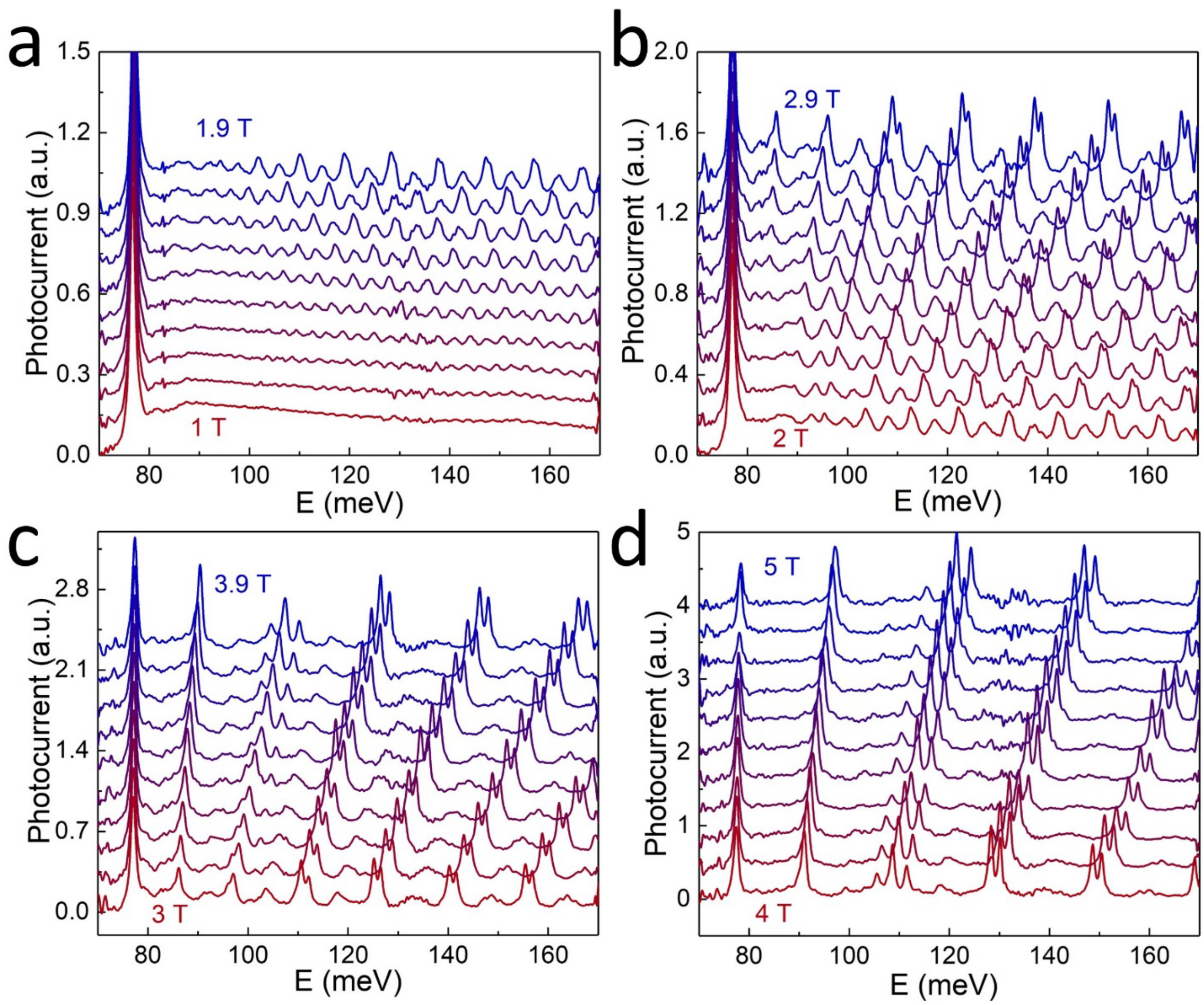

Supplementary Fig. 5 Photocurrent spectrum of BLG in the magnetic field range of 1-5 T and *D* = 0.874 V/nm. In each plot, the spectra are shifted along the y-axis for clarity, and each adjacent pair of spectra are taken at magnetic fields different by 0.1 T.

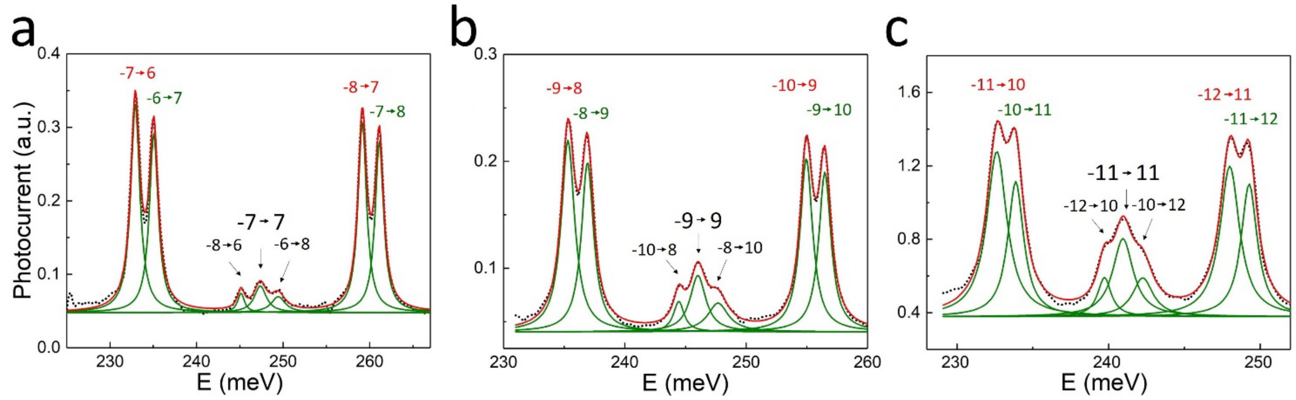

Supplementary Fig. 6 Extracting oscillator strength of inter-LL transitions using multiple Lorentzian fitting at *B* = 3.8 T. Here we show three examples of fittings for *D*= 1.32 V/nm. In

each plot, the original data is plotted in the black dotted line, seven Lorentzian peaks are plotted in green solid lines and the sum of them is plotted in red solid lines.

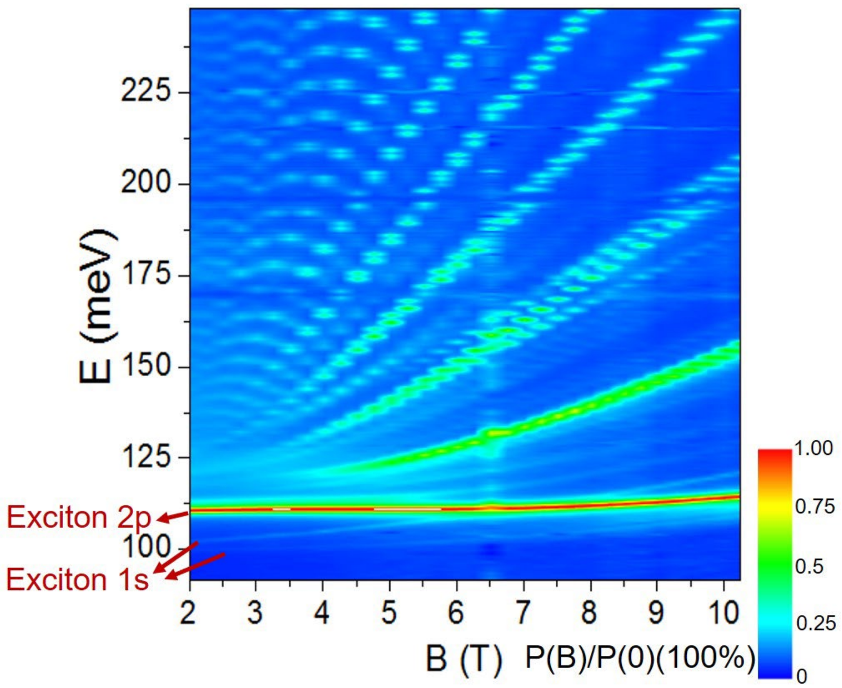

Supplementary Fig. 7 A 2D color plot of photocurrent spectra P(B) in the range of 2-10.25 T normalized by P(0) at 0 T and *D* = 1.32 V/nm. All features are qualitatively the same as that in Fig. 1d in the maintext. The split 1s exciton peaks are clearer since they are now in the transparent range of ZnSe lens.

Supplementary References:

1. McCann, E. & Koshino, M. The electronic properties of bilayer graphene. *Reports on Progress in Physics* **76**, 056503 (2013).
2. Koshino, M. & McCann, E. Landau level spectra and the quantum Hall effect of multilayer graphene. *Physical Review B* **83**, 165443 (2011).
3. Neto, A.C., Guinea, F., Peres, N.M., Novoselov, K.S. & Geim, A.K. The electronic properties of graphene. *Reviews of modern physics* **81**, 109 (2009).
4. Goerbig, M. Electronic properties of graphene in a strong magnetic field. *Reviews of Modern Physics* **83**, 1193 (2011).
5. Falkovsky, L. Quantum magneto-optics of graphite with trigonal warping. *Physical Review B* **84**, 115414 (2011).
6. Li, Xiao. Quantum Hall effects in novel 2D electron systems: nontrivial Fermi surface topology and quantum Hall ferromagnetism. PhD diss., 2014.
